# Effects of Fumed Silica on Thixotropic Behavior and Processing Window by UV-Assisted Direct Ink Writing

**DOI:** 10.3390/polym14153107

**Published:** 2022-07-30

**Authors:** Fengze Jiang, Mingyong Zhou, Dietmar Drummer

**Affiliations:** 1 Institute of Polymer Technology (LKT), Friedrich-Alexander-University Erlangen-Nuremberg, Am Weichselgarten 10, 91058 Erlangen, Germany; dietmar.drummer@fau.de; 2 College of Mechanical and Electrical Engineering, Central South University, Changsha 410083, China; mingyong.zhou1989@csu.edu.cn

**Keywords:** rheology, thixotropic behavior, UV curing, direct ink writing, additive manufacturing

## Abstract

In this research, the effects of fumed silica (FS) on the Ultraviolet (UV)-ink rheological behavior and processing windows were discussed. Objects using different concentrations of FS inks were printed by the modified UV-Direct ink writing (DIW) printer. The function of fumed silica in the ink-based system has been verified, and the processing scope has been expended with a suitable amount of FS combined with the UV light. The results show that the combination of a suitable amount of FS with the UV-DIW system reaches fast and accurate printing with a larger processing window compared to the non-UV system. However, an excessively high concentration of FS will increase the yield stress of the ink, which also increases the requirement of extrusion unit and the die-swelling effects.

## 1. Introduction

UV curing additive manufacturing (AM) has been extensively used since its first invention by Hull in 1988 [1]. After that, different kinds of UV curing AM methods were invented to fast manufacture prototypes and demonstrators [2,3,4]. Until recent decades, researchers have attempted to transfer stereolithography from mere prototypes to mass production, which led to another popular trend and generated numerous companies focusing on these targets [5,6,7].

Fumed filament fabrication (FFF) is normally considered as melting the filament material that extrudes from the metal nozzle [8,9]. The easy molding and harmless filament material, for example, polylactic acid (PLA), has been widely used worldwide, especially in the desktop market [10,11]. Various materials are used in different applications, including highly filled composite materials and metal powder composite filament [12,13,14]. However, the melting process has a theoretically maximum extrusion speed during the heat transfer, limiting the printing speed [15]. Increasing the extrusion temperature will cause other geometric accuracy issues because of the requirement of a longer cooling time [16]. On the other hand, the self-cooling system also limited the applications for complex structure printing; only precise adjustment of parameters enables high-quality complex structures. The adhesion between different layers of filament is based on the fusion of the semi-melting status of the materials [17]. For better support, most of the filament set the temperature near glass transition temperature, which limited the bonding strength between layers.

Digital light processing (DLP) printing methods have improved tremendously in recent years, and from the very beginning, it was implemented as a faster alternative to Stereolithography (SLA) with smaller size, which is better for desktop usage [3]. With the improvement of the technology, the accuracy is based on high-resolution projector or liquid crystal display (LCD) screen [18]; the special dead zone design and other related fast cooling DLP systems generate a massive market for both industrial and home usage [19,20]. The liquid resin system meets the requirement of particle system, which has been widely used for printing ceramic parts. There are existing branches of research on ceramic ink printing and sintering after post-curing [21,22]. However, the increased content of ceramic powders dramatically increases the viscosity and limits the penetration of the UV light, which leads to a decrease in the maximum curing depth and the decrease in the printing efficiency of the same model [23,24]. As additive manufacturing gradually transfers from prototyping to a ready-to-use manufacturing method, the requirement of mechanical properties has increased more than before. However, in the vat stereolithography, short fiber orientation is difficult to control, let alone the continuous fiber reinforcement, which is the most common path to improve the mechanical properties of the polymer parts [25,26,27].

Hybrid printing is one of the most attractive methods since it combines several advantages of different technologies. UV-DIW has a wide selection of ink materials, and the extrusion-based printing methods are adaptable to many filler-containing systems, including carbon fibers, silicon rubber, hydrogel [28] and even metal powders [29]. With the assistance of the six-degree freedom robot arm, the extrusion ink with suitable viscosity generates complex 3D structures instead of the regular additive manufacturing that only prints layer by layer [30].

Although UV-DIW printing has elicited more attention, some detailed fundamental knowledge has not been clearly discussed yet. In this study, the effects of fumed silica amount on the ink thixotropic behavior, related yield stress and printability have been extensively discussed. With the assistance of UV light, in comparison with the regular DIW process, the minimum viscosity is decreased together with the broadening of the processing window due to the fast curing process. The smooth flow ink also increases the potential of composites with a higher filling degree for both geometry accuracy and printability.

## 2. Materials and Methods

### 2.1. Ink Formulation

One of the most important parts of UV-assisted direct writing is the formulation of the ready-to-print ink that meets the requirements of suitable curing kinetics, viscosity, and shear thinning properties to provide the possibility of different applications, including the high filled particles, fast printing and complex structures. In this case, it is required that the ink has the properties with sensitive flow responses which perform the non-Newtonian shear thinning and thixotropy upon the imposition of shear stress. To attain the abovementioned properties, adding thixotropy agent into the formula is of necessity. Fumed silica (FS) with an average particle size of 20 nm is a widely used agent as a viscosity adaptor, and especially as the thixotropy agent [31].

The thixotropic behavior is essential to maintain the shape of the printed filament after extruding from the tip, which facilitates the printed part to preserve its shape before curing.

To impart elasticity to the ink, a percolating network of FS needs to be formed once exposed to the resin solution. This was accomplished by the addition of hydrophobic FS to more polar resins or, conversely, hydrophilic FS to less polar formulations. The ink formulations contain the following main components: polyurethane acrylate (PUA) oligomers, reactive diluents, photoinitiator, and a rheology modifier. The concentrations of the individual components of the inks varied to obtain the specific functionalities desired in each printed object. The PUA oligomers that polymerized into either soft or hard networks were used in different proportions as base ingredients of all the inks. The reactive diluents were further added to alter the mechanical properties of the inks after consolidation [14].

The oligomer used for the customized UV curing resin is aromatic urethane acrylate (Photomer 6628), which is ideal for adhesives and applications for inks formulation, with a viscosity of 80 Pa·s at 25 °C. The monomer is 1,6-Hexamethylene diacrylate (HDDA, Photomer 4017), with a viscosity of 5–10 mPa·s at 25 °C. Inhibitor, with a concentration between 100 and 500 ppm, has been pre-added into the monomer. The photoinitiator 2-Hydroxy-2-methyl-1-phenylpropanone (Omnirad 1173) was prepared with the concentration of 3 wt%, which has distinguished absorption peaks at 244 nm and 330 nm. The formula of the ink is presented below in Table 1 [32].

### 2.2. Preparation of UV Curing Ink

The centrifugal mixer Thinky ARE-310 (Thinky Inc., Laguna Hills, CA, USA) was used to uniformly mix the main UV resin components mentioned above with different amounts of fumed silica at 2000 rpm for 10 min. All materials were purchased from IGM Resins, the Netherlands. Fumed silica was purchased from Evonik AG and was used as received. 

The preparation of inks begins by mixing the oligomer and monomer to a certain viscosity; half of the FS powder was added into the ink because the relatively low density of FS leads to large volume. To prevent any agglomeration, the second half of the FS powder was added into the ink after the first half of the FS powder was uniformly mixed. The abovementioned steps were repeated after the introduction of all the powder. The heat was generated by the friction immediately after the preparation while the shear stress remained. Thus, the ink maintains a liquid form. Subsequently, the ink was poured into a 20 cc syringe which was covered with aluminum foil and prepared to be printed.

### 2.3. Rheological Properties Measurement

Rheological measurements were carried out in a stress-controlled rheometer DHR-2 (TA Instruments, New Castle, DE, USA), using the measuring geometry of serrated parallel plates with a diameter of 20 mm. Measurements were carried out at 30 °C. For all the rheological experiments, a 1000 µm gap was used without pre-conditioning shearing treatment for the tested inks [33]. The stress-controlled oscillatory measurements, which were performed at 1 Hz, were used to obtain storage and loss modulus. After loading and trimming the edge of the samples, the samples were allowed to relax for 5 min before measurement. During the measurement, the increase of the applied stress was proceeded in a stepwise manner until exceeding the yield stress of all samples, and the crossover point of the storage and loss modulus was defined as the yield stress for different ink formulations. The steady-shear viscosity of the inks with maximum distinct shear rate up to 1000 s^−1^ was collected in another set of experiments. The thixotropic property measurement program is shown in Table 2.

The mixed resin was measured under both flow and oscillation rheological tests to collect related information.

Stress sweep analysis during oscillatory measurements was carried out with logarithmically increase of shear stress from 10 Pa to 1000 Pa at a fixed frequency of 1 Hz based on the previous pretesting. Maximum storage and loss modulus in the linear regime were evaluated and compared in parallel to the printability. The stress at which the G′ and G″ crossed was taken as the gel point. Each sample was measured in triplicate.

### 2.4. UV-Assisted Direct Writing Setup

The prepared ink was filled into a 10 mL commercial syringe using a flat needle with the inner diameter at 0.84 mm and was then set up to a self-modified printer.

A self-modified 3D printer (Ender 3 Pro, Shenzhen, China) was used to perform printing experiments. Printing was carried out under ambient temperature. A printing design of a hollow square column with a bottom dimension of 30 × 30 × 10 mm and wall thickness of 0.84 mm was created using Slic3r open-source software. A UV-blocked tip with the inner diameter of 0.84 mm (18G) was used as extrusion nozzles. Printing experiments were carried out with the nozzle using a printing speed of 25 mm/s with a single-layer sidewall, while the printing layer height was set to 0.5 mm. 

**Figure 1:**
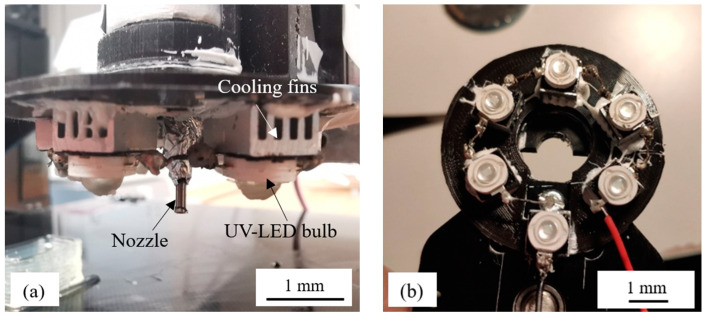
(**a**) Nozzle region design; (**b**) distribution of six UV lights.

To provide the necessary irradiation for polymerization, six UV LED lights with the wavelength of 365–370 nm were fixed at the opening of the nozzle tip, as shown in Figure 1a. The lights were fixed on the aluminum cooling fins to dissipate the heat and the nozzle covered by aluminum foil to avoid light leakage. The distribution of the light has an overlapping region which is located at the nozzle tip. The light intensity was measured by radiometer which was set as 1 mW/cm^2^, as shown in Figure 1b, and was fixed during the whole process.

**Figure 2:**
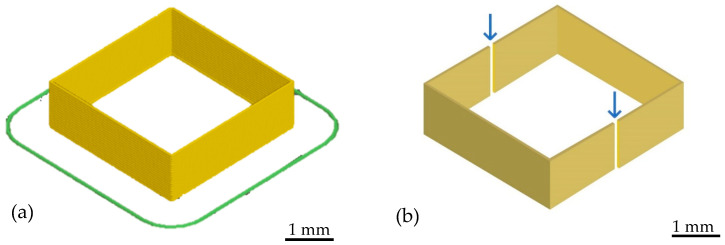
(**a**) 10 mm height cube model; (**b**) cross-section location of the microscopy photos.

To find out the effects of the FS amount on the samples quality, a simple cube model was used and sliced with the software. The cube has both the length and the width of 30 mm, and the height of 10 mm together in 12 layers, as shown in Figure 2a.

### 2.5. Morphology

The morphology of the printed cross section was characterized by incident light microscopy Axiophot (Carl Zeiss, Jena, Germany). The samples were cut from the middle part of the cube, as shown in Figure 2b. To prevent cracking, the samples were buried in the epoxy resin to strengthen the structure. The layer structure and the voids were observed during the process.

## 3. Results and Discussion

### 3.1. Rheological Behavior

**Figure 3:**
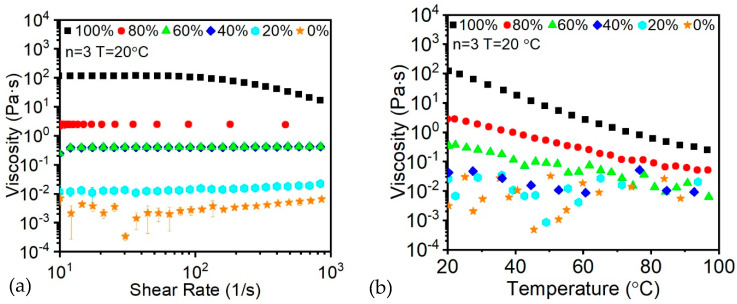
Oligomer ratio—related (**a**) shear thinning; (**b**) temperature dependent viscosity.

To begin with, the pristine oligomer and monomer resin without FS were tested for the shear thinning effects. Given the initially low viscosity of the oligomer and monomer resin in the presence of diluent, only 100% oligomer exhibits a significant shear thinning effect, as shown in Figure 3a. Moreover, the average viscosity was exceedingly low for the extrusion-based printing usage, while risking instability after printing.

However, from Figure 3a, it is shown that with the increasing of viscosity, the shear thinning effect remains unchanged, which causes difficulties during extrusion and increasing the requirement of the motor torque. As a consequence, the addition of thixotropy agent was selected to overcome the issue. Also, since the UV curing process is an exothermal reaction, the increasing of ink temperature will also decrease the viscosity of the ink that will lead to even more instability of the printed part. In this case, a temperature ramp of the resin has been taken, as shown in Figure 3b.

### 3.2. Thixotropic Properties

**Figure 4:**
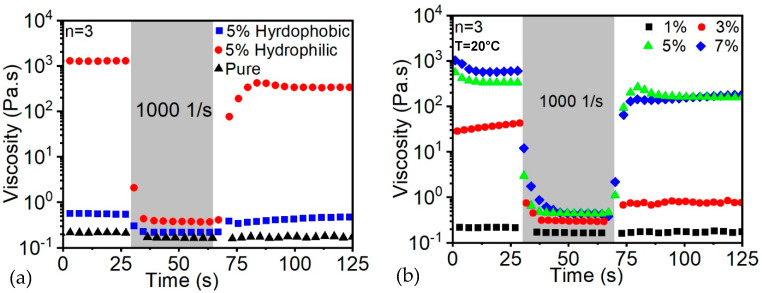
The effects of (**a**) two types of FS; (**b**) FS amount on ink thixotropic behavior.

The thixotropic properties describe the reformation or rebounce close to the original properties after the removal of shear stress. With the increase of thixotropic properties, the stability of the sample after being extruded from the nozzle together with the maintenance of the accurate geometry are significantly improved. To increase the initial viscosity and the relative thixotropic properties, the fumed silica was integrated into the resin. By collecting rheological data, the steady-state viscosity of the inks were quantified, as shown in Figure 4a. The result shows that in the absence of FS, the base resin exhibits a Newtonian response with a viscosity of 1 Pa·s, while with the addition of 5 wt% FS, the viscosity reached up to 104 Pa·s.

In our resin system, the hydrophobic FS was not a suitable agent since, after a short period of time, precipitation of a thin layer was formed at the bottom of the resin container. Moreover, the static viscosity was merely increased, which is similar to the addition of filler inside, as shown in Figure 4a.

In contrast, the hydrophilic FS tremendously enhances the initial viscosity of the resin together with the thixotropic properties of the ink. Thus, the ink was easily extruded from the nozzle without a strong motor. In addition, when the shear stress was removed, the viscosity resiled to around 90% of the initial viscosity in a few seconds. The observations of hydrophilic FS were of immense importance for the ink in order to remain stable after being extruded from the nozzle.

The effect of the addition of FS on the rheological behavior of an exemplary resin composition was studied through oscillatory measurements, as shown in Figure 4b. With the increase of FS particles incorporated into the base resin, the rheological behavior of the fluids follows the pure Newtonian behavior to a viscoelastic behavior with distinct yield stress. Specifically, the increment of FS content from 1% to 7% demonstrates an increase of the yield stress together with low-shear viscosity of the resin by two orders of magnitude. Distortion of the printed objects was prevented due to the essential viscoelastic nature of the inks with high loading of FS. Since the thickening of the fluid does not require the participation of chemical reaction for the rheological modifier, a series of resins including epoxies, silicones and acrylics were successfully printed using the exemplary FS resin composition. Thus, the abovementioned ink system establishes a useful platform with a library of selected materials suitable for the UV-assisted direct writing of objects with controlled orientation, composition and shape.

### 3.3. Yield Stress Behavior

**Figure 5:**
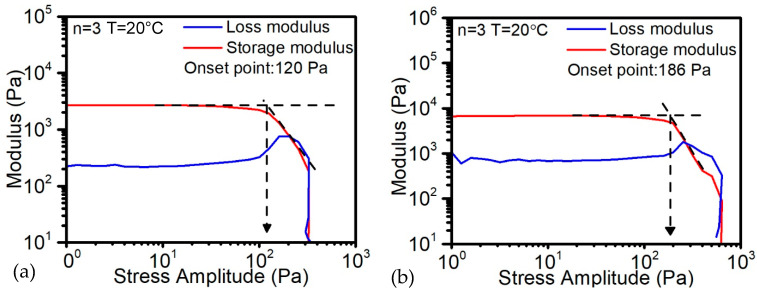
Dynamic modulus and yield stress of (**a**) 5 wt% FS; (**b**) 7 wt% FS ink.

In order to prevent the shape distortion of objects with 3D geometries and the extrusion pressure, the yield stress of ink was investigated with increasing contents of the thixotropic agent for tracking potential changes in structural geometry after a fixed period of time. The results show that when the FS amount is lower than 5%, the ink still performs liquid status, which indicates that the loss modulus is higher than the storage modulus. When the FS amount arrived at 5%, from Figure 5a, the storage modulus reached 2800 Pa while the yield stress is 120 Pa. With the increasing of FS to 7%, the storage modulus increased to 6600 Pa and the yield stress increased to 186 Pa, as shown in Figure 5b. In this case, the higher amount of FS will raise the requirement of the extrusion stepper motor to extrude the material smoothly.

### 3.4. Ink Printability and Sample Stability

**Figure 6:**
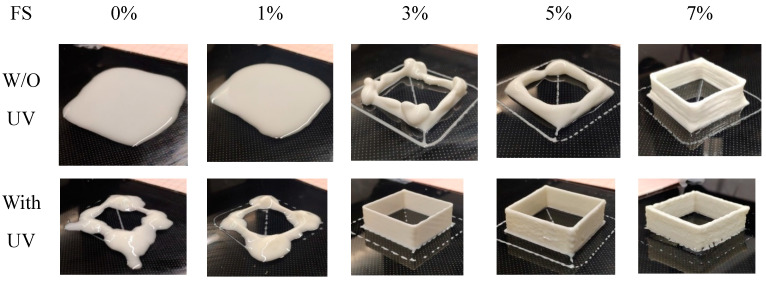
Printability with different amounts of fumed silica with and without UV light.

Figure 6 shows the printed samples of different solid compositions. From left to right, the FS amount gradually increased from 0% to 7%, respectively, and the ink with lower than 3 wt% loadings was insufficient to maintain the geometry of the part due to the relatively low elastic modulus. The ink is directly spread on the substrate. In the presence of UV light, the central part of the cube is discovered, while the pause originated from changing directions of the nozzles concentrated the major ink at the corner. With the addition of 3% of FS, several layers of the cube were generated, supported by its inherent strength; however, the disintegration occurs once, reaching about five layers. In the case of 5% FS loading, the shape of the cube is more clear, and once it reaches 7%, the pronounced and accurate structure of the cube is generated with a sharp edge that reflects the non-shear viscosity of a good condition for self-support. However, with the assistance of UV light, the cube can be printed in good condition since 3% of the FS. These results show that the combination of the extrusion—based printing and UV light enables the broadening of the process window. 

Meanwhile, the thickness of the wall is discovered to exhibit an obvious increase with the enhancement of FS content. Moreover, under different extrusion conditions, the thickness of the wall is enlarged in comparison with the original model. Table 3 shows the measured wall thickness at the center uniformed region by calipers. With three times measurement, it was shown that, when increasing the amount of FS, the wall thickness has an increasing tendency compared to the designed thickness. While the 3% FS is a little smaller than the designed 0.84 mm thickness, the 7% FS ink dramatically increases the thickness to 104% percent. This increment clearly decreases the printing accuracy of the part. The reason for the phenomenon is because the increment of FS increases the yield stress of the ink, which increases the die-swelling effect, and, due to the extruded extra volume of ink, the surface quality also decreased. 

### 3.5. Morphology of the Cube Structure

**Figure 7:**
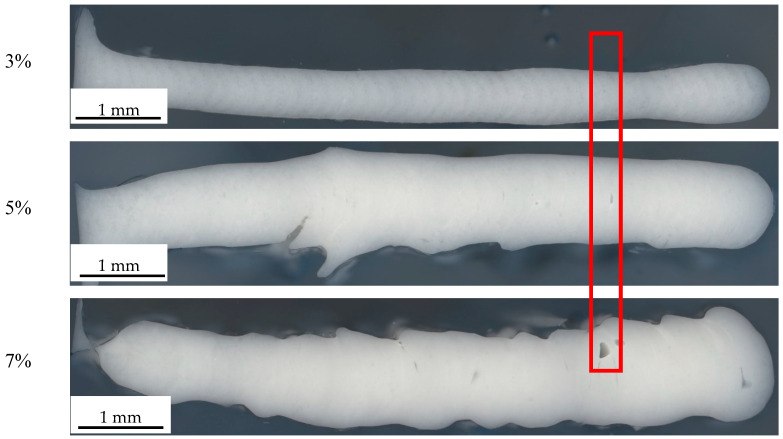
Microscopy image of the vertical cross section of the cube.

Since the clear differences appeared on the successfully printed cubes, optical microscopy images were taken to observe the differences from the 50× magnification structures, as shown in Figure 7. It is more obvious that, with the increase of the FS loading, the wall thickness is dramatically increased. Besides the wall thickness, the existence of voids and unfilled layer structures are observed due to the enhanced FS loading that increases the yield stress and related die-swelling effect. Whereas, the elevated yield stress requires higher pressure from the nozzle, which makes the shear rate near the critical point, leading to unstable printing. Because of the semi-curing status of the ink before contact with the UV light, there is still some time to let the ink flow on the printed surface. In this case, the cross section presents a smooth surface based on the fusion between different layers. The larger magnification image can be found in Figures S2–S4.

**Figure 8:**
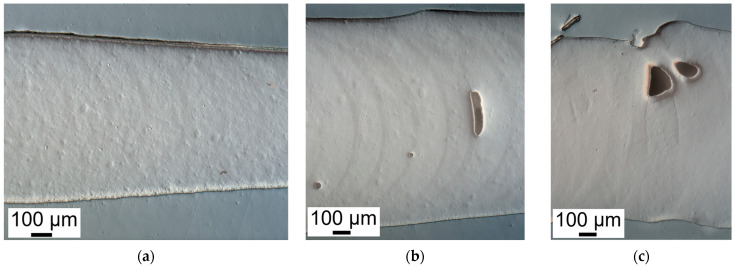
Darkfield microscope image of (**a**) 3%, (**b**) 5%, and (**c**) 7% FS.

Darkfield images were taken at the red box region to better observe the layer features, as shown in Figure 8. With the increment of FS, the meniscus curve at each layer gradually appeared on the images. Also, because of the higher thixotropic property, the flowability of the resin decreased. The void started to generate, which indicated that the ink was cured before it could fill in the cavity. 

**Figure 9:**
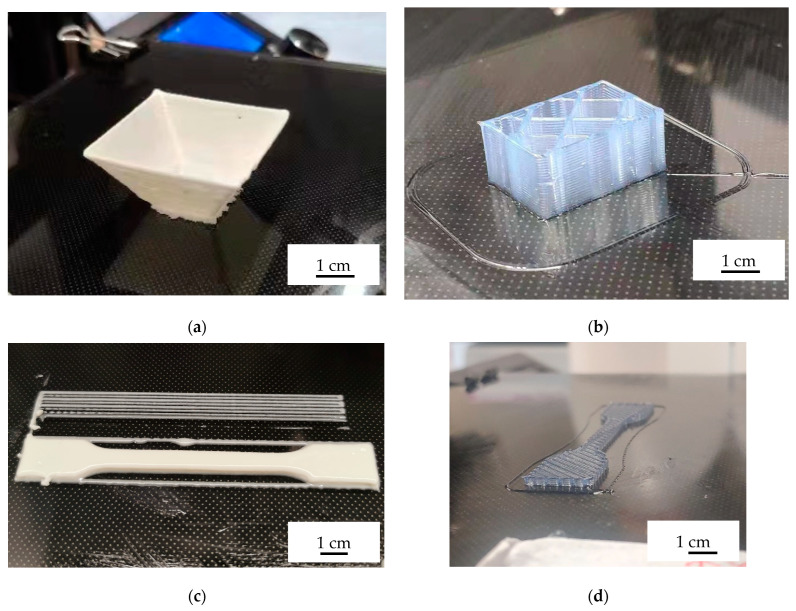
(**a**) 45-degree square frustum; (**b**) cube with reinforcement ribs; (**c**) solid-filled tensile bar; (**d**) hollow tensile bar with 45° crossover directions.

With the expanded processing window, more multipurpose structures were manufactured to demonstrate the possibilities in Figure 9. The structures show the potential of UV-assisted direct ink writing.

## 4. Conclusions

In this paper, the effects of thixotropy agent amount on the printability of the UV-DIW were discussed. The requirements of the printing system were adjusted by changing the concentration of FS. The results show that, with the increment of FS, not only the non-Newtonian behavior is strengthened including shear-thinning and thixotropic behavior, but the ink yield stress and static viscosity also increased, which is critical to maintaining the geometric accuracy of the printed samples. However, the extra FS also leads to higher extrusion pressure and more extrusion swelling during the printing process. In this case, the samples have a pronounced and larger diameter compared to the nozzle size, which should be avoided. A comparison of how the FS affects the printability of the samples is also discussed; the samples with and without UV light clearly show how the FS improves the printing process and also show how the UV light expands the processing window compared to a non-UV system.

The results of this paper provide guidance for material design and development for the UV curing ink based on different applications in terms of printability. Some different structures presented the possibilities of the technology. The investigations for the application of FS provide information for regular ink printing and composite ink printing.

## Figures and Tables

**Table 1:** Ink formulation in the experiments.

FS Ratio	FS Weight (g)	Oligomer (g)	Monomer (g)	Photoinitiator (g)
0%	0	11.4	8	0.6
1%	0.2			
3%	0.6			
5%	1			
7%	1.4			

**Table 2:** The thixotropy behavior program.

Program	Shear Rate (1/s)	Time (s)
Conditioning sample	0	60
Flow Peak	0.1	5
Flow Ramp	0.1–1000	30
Flow Peak Hold	1000	5
Flow Ramp	1000–0.1	30
Flow Peak Hold	0.1	90

**Table 3:** Wall thickness of successfully printed cube.

FS Amount	Wall Thickness			Average	Ratio
3%	0.8	0.8	0.8	0.8	−5%
5%	1.3	1.5	1.45	1.41	+67%
7%	1.55	1.7	1.9	1.72	+104%

## Data Availability

Not applicable.

## Supplementary Materials

The following supporting information can be downloaded at: https://www.mdpi.com/article/10.3390/polym14153107/s1, Figure S1: Microscopy image of the vertical cross-section of the cube; Figure S2: (a) The SEM and dark field images of the 3% FS cross-section; (b) the SEM and dark field images of the 5% FS cross-section; (c) The SEM and dark field images of the 7% FS cross-section; Figure S3: Effect of feed rate on printing continuity; Figure S4: Enthalpy of UV-curing resin undre different light intensity.
