# Supplementary material for: Effects of Fumed Silica on Thixotropic Behavior and Processing Window by UV-Assisted Direct Ink Writing
Source: Polymers (Basel). 2022 Jul 30;14(15):3107. doi: 10.3390/polym14153107 (PMC9370837; doi:10.3390/polym14153107)
Supplement: Supplementary file 1 [file polymers-14-03107-s001.zip › polymers-1790609-supplementary.pdf]

# Supplementary Materials

## Effects of fumed silica on thixotropic behavior and processing window by UV-assisted direct ink writing

Fengze Jiang[1,*], Mingyong Zhou[2], Dietmar Drummer[1]

1. Institute of Polymer Technology (LKT), Friedrich-Alexander-University Erlangen-Nuremberg, Am Weichselgarten 10, 91058, Erlangen, Germany
2. College of Mechanical and Electrical Engineering, Central South University, Changsha, Hunan 410083, China

*Corresponding author

(Phone: +49 9131 85-29736 Fax:+49 9131 85-29709, fengze.jiang@fau.de)

**S1. Extra microscope images**

Additional microscopy images were supplied including dark field and SEM. Since the images did not supply more information compare to the original microscope image. They were located in this part, where the voids could be found through the microscope.

3%

5%

7%

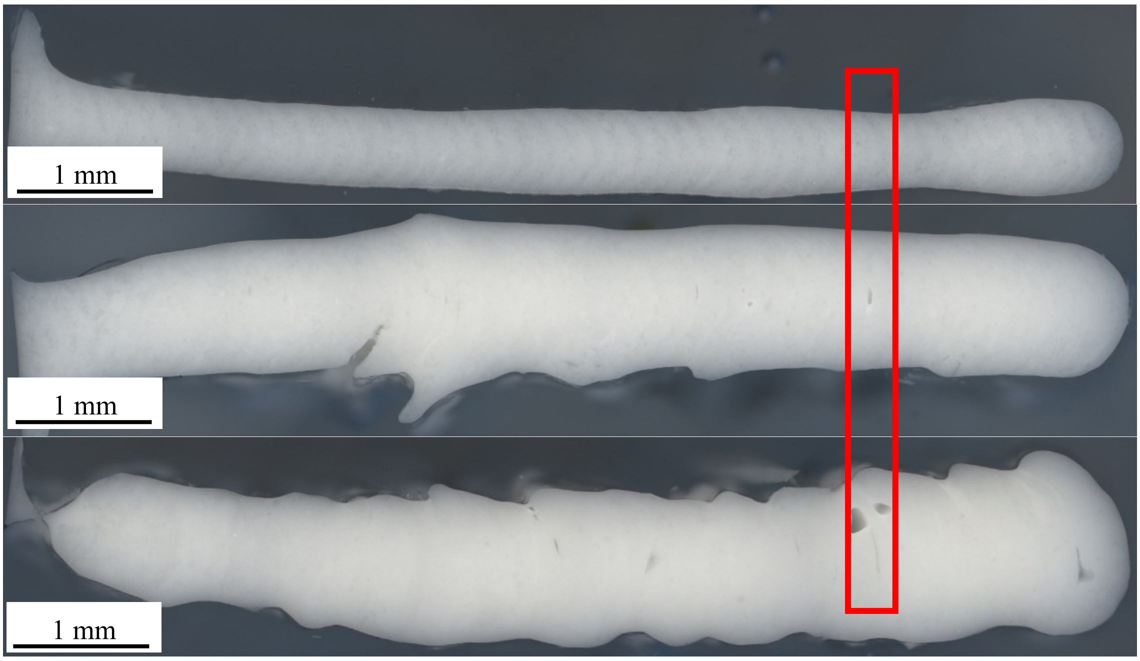


Figure S1. Microscopy image of the vertical cross-section of the cube.

SEM and dark field images of the cross-section of the cube under 100x magnification are shown in Figure S2.

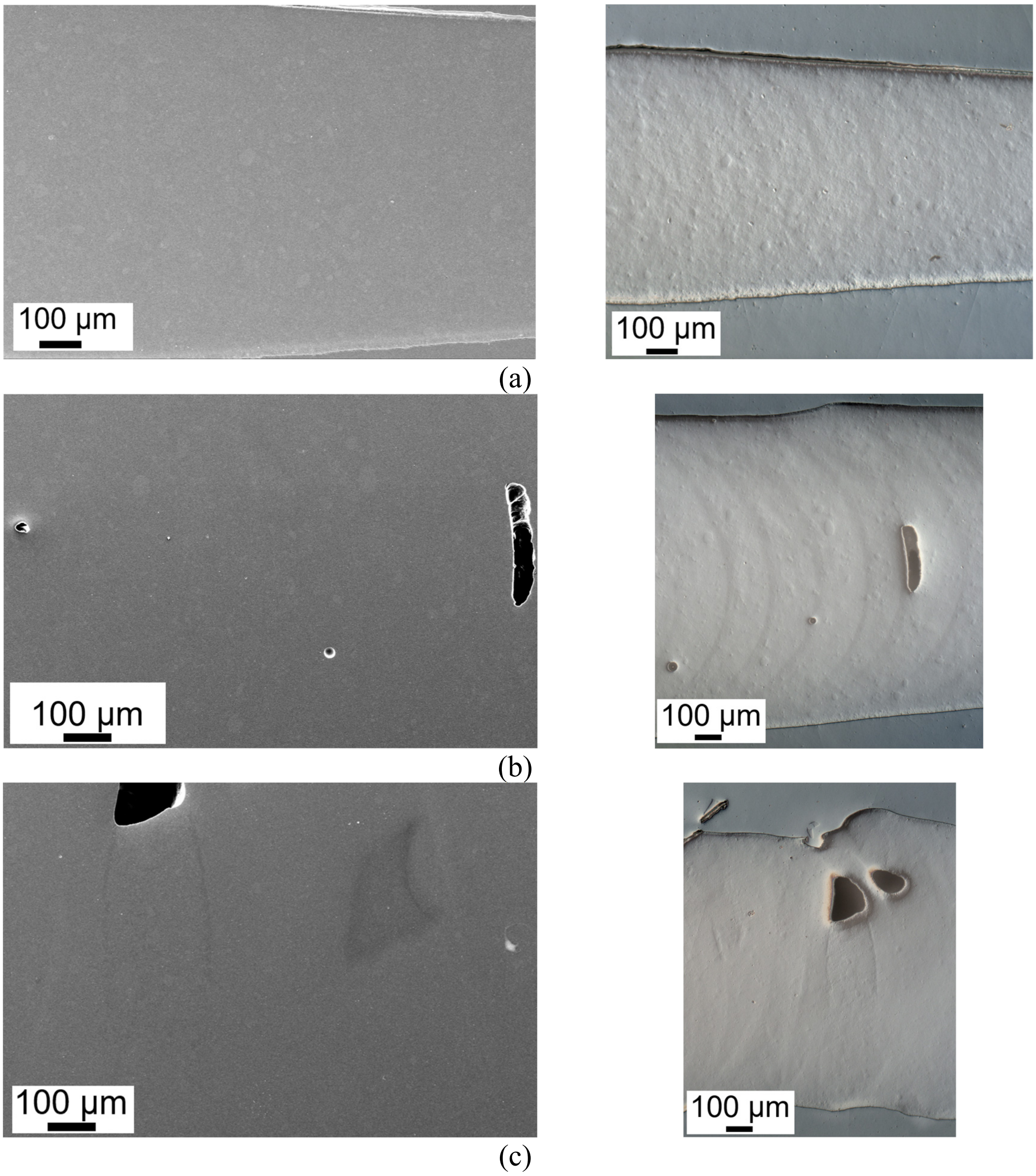


Figure S2. (a) The SEM and dark field images of the 3% FS cross-section; (b) The SEM and dark field images of the 5% FS cross-section; (c) The SEM and dark field images of the 7% FS cross-section.

**S2. Extrusion wire width (without curing)**

A standard 1cm cubic paper was fixed at the printing stage to measure the width of the printed lines. By counting cube numbers, we could collect the width information by the photos. Because of the limitation of the extrusion stepper motor, the increment of the ink initial viscosity will request a slower printing speed or longer reaction time to make the printing process stable. These results were designed to using different feed rate of the nozzle to test the limitation of the printers which could supply the information for further parameters setting. The colorful signs in the figure S3 shows the lines with different feed rate from 5mm/s to 65mm/s. The current stepper motor is NEMA17 with 65 N·cm holding torque.

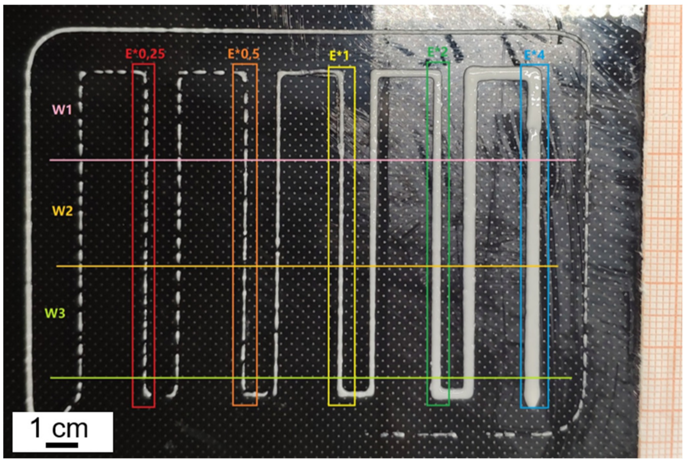


1 mm plotting paper as pixel ruler reference

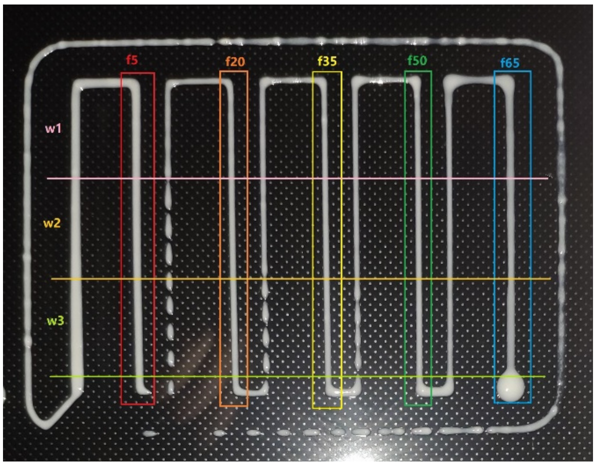


3% FS ink with five feed rate

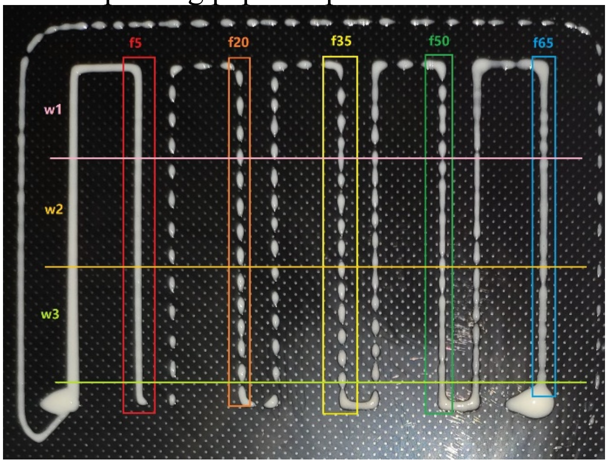


5% FS ink with five feed rate

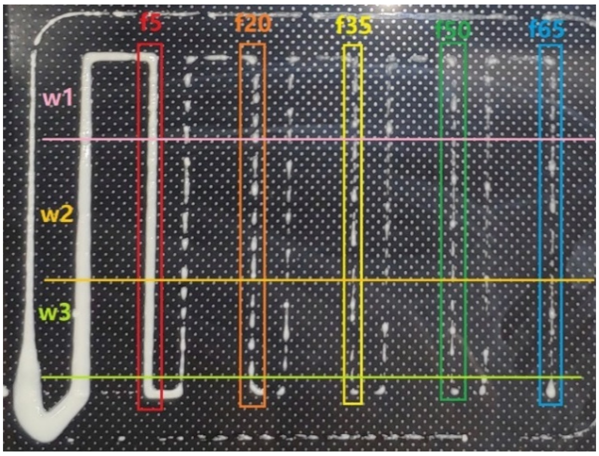


7% FS ink with five feed rate

Figure S3. Effect of feed rate on printing continuity.

**S3. Curing kinetics of the used ink**

The thermal property and the curing kinetics were measured by UV-DSC before. The enthalpy curves are shown in Figure S4:

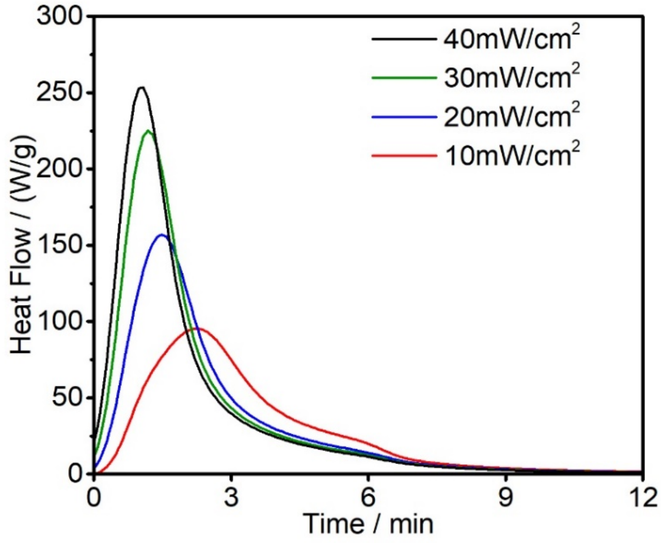


Figure S4. Enthalpy of UV-curing resin undre different light intensity.
